# Editorial: Polyoxometalates in Catalysis, Biology, Energy and Materials Science

**DOI:** 10.3389/fchem.2019.00646

**Published:** 2019-10-01

**Authors:** Soumyajit Roy, Debbie C. Crans, Tatjana N. Parac-Vogt

**Affiliations:** ^1^Eco-Friendly Applied Materials Laboratory, Department of Chemical Sciences, Materials Science Center, Indian Institute of Science Education and Research Kolkata, Kolkata, India; ^2^College of Chemistry, Central China Normal University, Wuhan, China; ^3^Department of Chemistry, Colorado State University, Fort Collins, CO, United States; ^4^Department of Chemistry, KU Leuven, Leuven, Belgium

**Keywords:** polyoxometalates (POMs), soft-oxometalates (SOMs), biology, catalysis, medicine, materials science

The chemistry of polyoxometalates/polyoxidometalates (POMs) continues to evolve and has resulted in Nobel prizes (Janell et al., 2001) and several technological advances (Hill, 1998; Cronin and Mueller, 2012; Galán-Mascarós and Kortz, 2018; Pope et al., 2019; Roy and Crans, 2016). The present Research Topic of Frontiers in Chemistry is a celebration of evolution of that ever-exciting chemistry. Though it is impossible to sample the growth of such an active area in the span of Research Topic of the journal Frontiers in Chemistry, it is still possible to give the reader a taste of where this chemistry is now and where it is heading. With this objective in view we have chosen four thematic topics in which this chemistry is evolving: Biology, Catalysis, Energy, and Materials Science. To chart the developments and to put the present trend in perspective, it is apt to archive the history of this field. Thus, to set the stage, arching back to the beginning, Kondinski and Vogt map the efforts of James Fargher Keggin and thereby connect the field to Bragg and Pauling as well as to modern times, showing how structural studies have shaped this field and its developments over time to date. Thereafter, we continue our journey with catalysis and energy harvesting with a review by Sullivan et al. highlighting POM-containing multi-hydrogen-bonding polymers that undergo gelation upon exposure to select organic liquids and simultaneously catalyze reactions along with their use in photoelectrocatalytic water splitting for energy harvesting. A third review by the Nowak group (Wawrzynczak et al.) outlines different catalytic applications of polyoxoniobates in water oxidation, hydrogen evolution, and allied reactions and gives a comparison with niobium containing mesoporous silicates. The Patzke and Galan-Mascaros group (Soriano-López et al.) show pre-catalytic incorporation of the cationic photosensitizer into the anionic polyoxometalate as in RuCo_9_, which significantly improves oxygen evolution. Such an effect elegantly demonstrates facilitated electron transfer due to photosensitizer-polyoxometalate pairing in solid-state, enhancing the long-term stability of the photo-sensitizer. By coupling water-oxidation with CO_2_ reduction, the Roy group (Das et al.) shows that using Muellerene type {Mo_368_} giant polyoxometalates, efficient energy harvesting homogenous catalytic systems can be realized. In another work, the Zhang and Wei group (Zeng et al.) show that nanospherical self-assembled Anderson type {CrMo_6_} polyoxometalate and pillar[5]arenes can oxidize aldehydes to carboxylic acids.

As we move from Catalysis to Materials Science, we come across the one pot synthesis of a magnetic polyoxometalate containing one or two subunits of [Co^III^${\text{Co}}_{3}^{\text{II}}$O_4_] cubane by the Coronado group (Duan et al.). In another work the Hayashi group (Sugiarto et al.) uses Cobalt as four [fac-{Co(tacn)}^3+^] units capping a distorted cubane-like V_4_O_4_ to contain the growth of polyoxometalate with far-reaching consequences in studying and trapping of synthetic intermediates in POM research. The Mialane and Serier-Brault group (Salomon et al.) has in another work shown that incorporation of a luminescent europium polyoxotungstate into the cavities of a luminescent zirconium metal organic frameworks results in a multifunctional dual-luminescent material. The Prakash and Ramanan group (Tewari et al.) reports the synthesis and photoluminescent properties of two closely related series of lanthanide containing POM structures. From luminescence and magnetic properties, we move to supramolecular water clusters, which are reported by the Das group (Amanchi and Das) in the context of the synthesis and supramolecular chemistry of seven decavanadate containing compounds. In some of the crystal structures, the authors found that the non-covalent interactions between the lattice water molecules and metal coordinated water molecules lead to the formation of supramolecular water clusters. Can oxometalate architectures move and can such movement be observed? The Roy and Adhikari group (Mallick et al.) shows such motility in soft-matter states of oxometalates or soft-oxometalates exploiting the redox reaction on the surface of such states. From active matter we now move to biological matter, where POMs are extremely active.

Ranging from potential therapeutic agents for Leishmaniasis, photothermal therapy to medicines for diabetes and cancer, the issue explores various facets of POMs for biological applications. The collaborative efforts of the McLauchlan and Jones group (Dorsey et al.) demonstrate from the standpoint of vanadium concentration and speciation of decavanadate and orthovanadate as an inhibitor for phosphatases which translates to being effective as a therapeutic agent targeting the secreted acid phosphatase (SAP) from *Leishmania*. Furthermore, using ^51^V NMR studies the Crans group (Samart et al.) shows that from the analysis of speciation in oxo-vanadate solutions, decavanadate is a more potent growth inhibitor of two Mycobacterial strains than other oxovanadates. The activity of decavanadates against diabetes is further explored by the group of González-Vergara (Sánchez-Lara et al.), who show synthesis and structural features of decavanadate salts of Cytosine and Metformin as a potential metallodrug against diabetes and cancer. In a very detailed study, (Gumerova et al.) explore the antibacterial activity of a set of 29 POMs against *Moraxella catarrhalis*. For these bacteria, the Preyssler type POM and the Dawson-type POM were found to be most potent oxometalates and surpass the activity of the Keggin and Anderson types of POMs. Covalently modifying a Keggin type POM, the group of Bonchio and Carraro (Zamolo et al.) obtains a bis-biotinylated conjugate with Avidin to show “selective targeting of proteins by hybrid POMs.” The group of De Matteis and Mitchell (Artiga et al.) achieves cellular internalization of gold nanorods inside a biocompatible and cell-adhesive chitosan hydrogel matrix using Keggin-type polyoxometalate (POM) as an inorganic gelator for “effective *in-vitro* photokilling.” In a work that brings biology, materials science, and catalysis closer, the group of Parac-Vogt (Quanten et al.) explores how selectivity and reactivity of Ce^IV^ and Zr^IV^ substituted Keggin type POMs are affected by various surfactant solutions in the hydrolysis of Cytochrome c, a α helical protein with haem group. With the aid of a series of experiments, the authors show the role of surfactant in facilitating the unfolding of Cytochrome c and in turn dictates the ease of hydrolysis of the protein. With all these works the science of polyoxometalates becomes a melting pot where biology, soft-matter, material science and catalysis science meet, and this Research Topic of Frontiers in Chemistry captures part of the progress in this area.

It has been a privilege for us all as editors working with all the contributors and reviewers in curating the Research Topic, and we thank all—including the Frontiers team—for making this journey a pleasant and timely one. We hope it will bring joy to readers and savor the examples of progress as they are nucleated together. It is our hope that the present Research Topic will ignite, initiate and illuminate the minds of many toward new and unexplored frontiers of polyoxometalate science.

## Author Contributions

SR, DC, and TP-V curated this special issue together.

### Conflict of Interest

The authors declare that the research was conducted in the absence of any commercial or financial relationships that could be construed as a potential conflict of interest.
